# Development and internal validation of a clinical prediction model for spontaneous abortion risk in early pregnancy

**DOI:** 10.1016/j.clinsp.2023.100318

**Published:** 2023-12-15

**Authors:** Junqing Li, Jimei Yang, Min Lv, Xiang Wang, Zhijing Chen, Na Zhou, Xuetao Hou, Zhen Song

**Affiliations:** a Imaging Diagnosis Department, Jinan Second Maternal and Child Health Hospital, Jinan City, Shandong Province, China; b Psychological Clinic, Jinan Second Maternal and Child Health Hospital, Jinan City, Shandong Province, China; c Clinical Lab, Jinan Second Maternal and Child Health Hospital, Jinan City, Shandong Province, China; d Department of Gynecology and Obstetrics, Jinan Second Maternal and Child Health hospital, Jinan City, Shandong Province, China; e Imaging Diagnosis Department, Jinan People's Hospital, Jinan City, Shandong Province, China

**Keywords:** Spontaneous abortion, Risk factors, Prospective study, COVID-19, Prediction model

## Abstract

**Objective:**

This study aimed to develop and internally validate a prediction model for estimating the risk of spontaneous abortion in early pregnancy.

**Methods:**

This prospective cohort study included 9,895 pregnant women who received prenatal care at a maternal health facility in China from January 2021 to December 2022. Data on demographics, medical history, lifestyle factors, and mental health were collected. A multivariable logistic regression analysis was performed to develop the prediction model with spontaneous abortion as the outcome. The model was internally validated using bootstrapping techniques, and its discrimination and calibration were assessed.

**Results:**

The spontaneous abortion rate was 5.95% (589/9,895) 1. The final prediction model included nine variables: maternal age, history of embryonic arrest, thyroid dysfunction, polycystic ovary syndrome, assisted reproduction, exposure to pollution, recent home renovation, depression score, and stress score 1. The model showed good discrimination with a C-statistic of 0.88 (95% CI 0.87‒0.90) 1, and its calibration was adequate based on the Hosmer-Lemeshow test (*p* = 0.27).

**Conclusions:**

The prediction model demonstrated good performance in estimating spontaneous abortion risk in early pregnancy based on demographic, clinical, and psychosocial factors. Further external validation is recommended before clinical application.

## Highlight

• A clinical predictive model for the risk of spontaneous abortion in early pregnancy.
• This model has added mental health factors compared to previous studies.
• Targeted interventions targeting high-risk women to reduce the risk of spontaneous abortion.

## Introduction

Spontaneous abortion, defined as the unintentional termination of a pregnancy before 20 weeks gestation, is a common complication affecting 10%‒15% of clinically recognized pregnancies.^1^ It can occur due to embryonic abnormalities, uterine abnormalities, endocrine disorders, infection, lifestyle factors, and other etiologies.^2^ Spontaneous abortion is not only a major pregnancy complication but also has significant psychological impacts on women. Several risk factors for spontaneous abortion have been identified in previous studies, including advanced maternal age, smoking, alcohol use, psychological stress, thyroid dysfunction, and polycystic ovarian syndrome.^3^, ^4^, ^5^ However, most existing studies utilize retrospective case-control designs and are focused on investigating individual risk factors rather than developing comprehensive prediction models.

There remains a need for robust and well-validated prognostic models that can estimate the risk of spontaneous abortion in early pregnancy based on multiple demographics, clinical, and lifestyle predictors. Accurate individual risk prediction can aid in counseling, monitoring and timely interventions for high-risk women to mitigate adverse outcomes. A few scoring systems have been recently developed to predict recurrent pregnancy loss rather than first-time miscarriages.^6^^,^^7^ However, those models have limitations such as small sample size (*n* < 500), inadequate validation, and suboptimal predictive performance (C-statistics < 0.7). Therefore, this study aimed to develop and internally validate a clinical prediction model to estimate the risk of first-trimester spontaneous abortion in pregnant women based on a wide range of predictors encompassing clinical, socio-demographic, lifestyle, and mental health factors.

## Materials and methods

### Study participants

Patients were recruited from January 2021 to December 2022. Baseline data was collected at the first prenatal visit. Follow-up of pregnancy outcomes continued until delivery. A total of 9,895 pregnant women were enrolled in this study, including 9,306 in the normal pregnancy group and 589 in the spontaneous abortion group. The inclusion criteria were established as follows: 1) Participants in this survey research were pregnant women who received an ultrasound diagnosis of normal intrauterine pregnancy and agreed to take part. 2) The study included pregnant women between the ages of 18‒48. Exclusion criteria were applied. 1) Pregnant women who have reproductive system abnormalities; 2) Pregnant women with autoimmune diseases, including antiphospholipid syndrome, systemic lupus erythematosus, undifferentiated connective tissue disease, Sjögren's syndrome, and others; 3) Patients diagnosed with severe heart, liver, kidney, and hematopoietic diseases; 4) Insufficient data collection in the cases studied.

### Study methods

Standardized questionnaires were developed in accordance with the survey plan, incorporating information on the age of the pregnant woman, gravidity, number of abortions, number of embryonic arrests, BMI, educational level, family income, history of hypertension, thyroid function, history of diabetes, history of polycystic ovary syndrome, assisted reproduction (if applicable), smoking and alcohol consumption history, exposure to pollution sources (including air pollution and radiation), frequency of staying up late, and recent home renovation status. The DASS-21 Chinese version was implemented to evaluate the psychological condition of expectant mothers. The scale comprises three subscales: depression, anxiety, and stress, with a total of 21 items. Patients utilized a 4-point scoring scheme ranging from “0” (disagree) to “3” (strongly agree) to indicate their emotional state during the past week, with elevated scores indicating more intense feelings. The DASS score was used to classify the measurement results into 5 levels: normal (depression score ≤9, anxiety score ≤7, stress score ≤14), mild (depression score 10∼13, anxiety score 8∼9, stress score 15∼18), moderate (depression score 14∼20, anxiety score 10∼14, stress score 19∼25), severe (depression score 21∼27, anxiety score 15∼19, stress score 26∼33), and extremely severe (depression score ≥ 28, anxiety score ≥ 20, stress score ≥ 34). The total scale achieved a Cronbach's α coefficient of 0.890, indicating strong reliability and efficacy for evaluating the mental health condition of pregnant women. Medical staff led pregnant women to scan the QR code and fill out personal information and questionnaires via smart devices during early pregnancy (within 6 weeks).

Regular ultrasound examinations were conducted to evaluate the embryonic health of patients. The spontaneous abortion group was identified as those who experienced spontaneous abortion due to embryonic arrest, while those who did not were classified as the normal group. A database containing information on patients with spontaneous abortion was created and reviewed by another researcher. The study protocol was approved by the Ethics Committee of Jinan Second Maternal and Child Health Hospital (Approval number: 2023-YBD-1-05). Prior to completing the questionnaire, the medical staff sought the opinions of the patients. Participation in the study required completion of the questionnaire. The researchers maintained strict confidentiality with regard to patients' personal information. As an observational study, this study follows the STROBE statement.

### Statistical methods

EpiData 3.1 and SPSS 27.0 (IBM Corp., USA) statistical software were used for data entry and analysis. The data was compared between two groups using T-tests and Chi-Square tests, and factors with statistically significant differences in univariate analysis underwent logistic regression analysis to screen out influencing factors of early spontaneous abortion. Multivariate regression analysis was performed on all possible predictive factors, and predictors with *p* > 0.05 were sequentially removed using multivariable logistic regression with backward stepwise elimination to identify independent predictors of spontaneous abortion. The results were considered statistically significant at a *p*-value of <0.05, and bootstrapping with 1000 samples was used to internally validate the model and adjust for optimism/overfitting. Discrimination was assessed by the C-statistic, and calibration was assessed using the Hosmer-Lemeshow test.

## Results

### Univariate analysis of general information and clinical factors

**Fig. 1:**
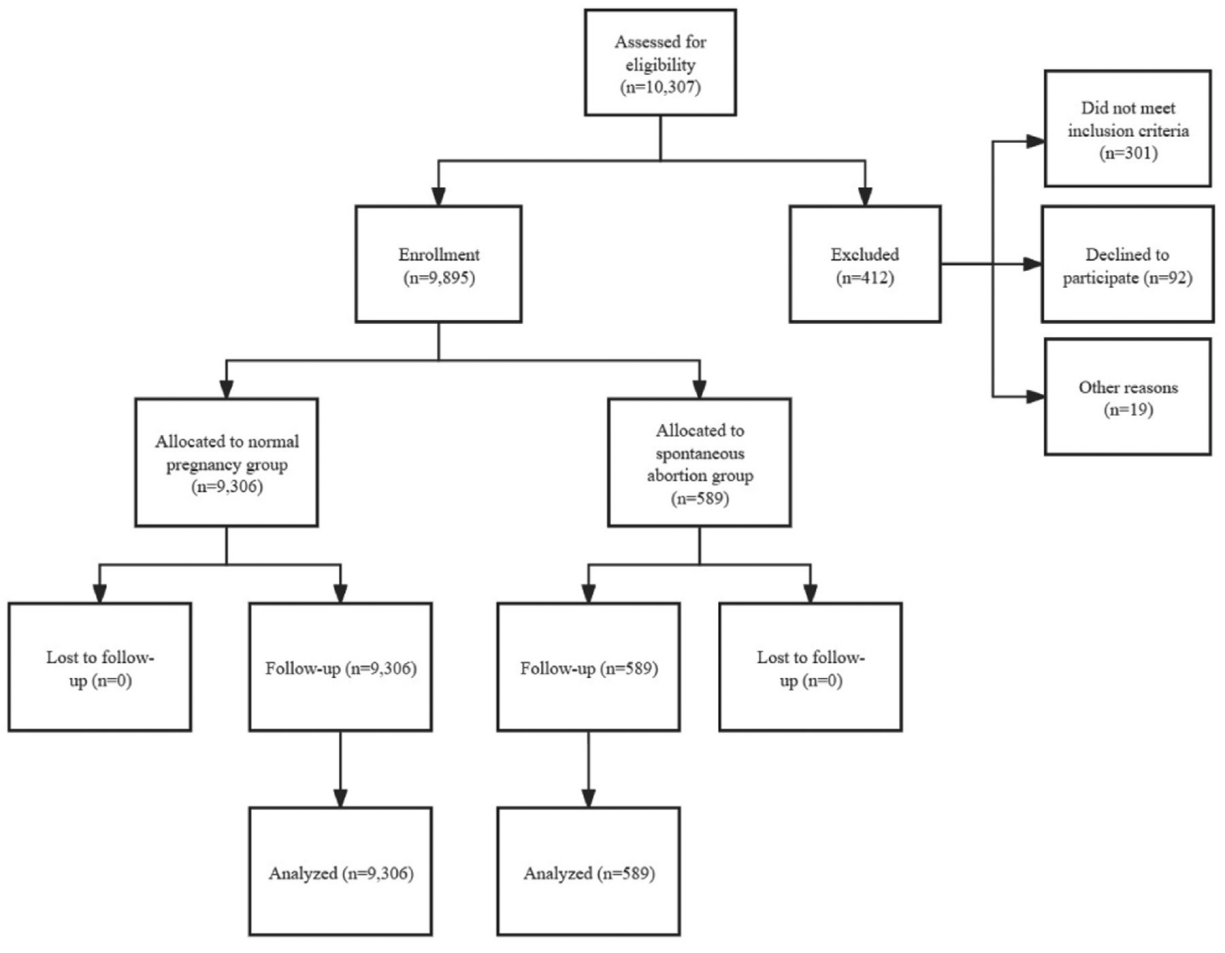
Flow diagram of study participants.

This prospective cohort study comprised 9,895 participants, with 9,306 in the normal pregnancy group and 589 in the spontaneous abortion group (Fig. 1). The mean age of the spontaneous abortion group was 33.03 ± 6.12 years, which was significantly greater than the mean age of 30.60 ± 5.98 years in the normal pregnancy group (*t* = 9.51, *p* < 0.05).

Univariate analysis of potential influencing factors indicates that the following factors have a significant impact on the outcome: BMI (χ^2^ = 9.13, *p* = 0.010), history of embryonic arrest (χ^2^ = 3427.87, *p* < 0.05), number of abortions (χ^2^ = 53.89, *p* < 0.05), thyroid dysfunction (χ^2^ = 19.05, *p* < 0.05), diabetes (χ^2^ = 7.32, *p* = 0.007), polycystic ovary syndrome (χ^2^ = 5.44, *p* = 0.02), assisted reproduction (χ^2^ = 34.47, *p* < 0.05), smoking (χ^2^ = 4.27, *p* = 0.039), and alcohol consumption (χ^2^ = 11.62, *p* < 0.05), there were considerable differences between the two groups in terms of exposure to pollution (χ^2^ = 8.84, *p* < 0.003) and recent home renovation (χ^2^ = 10.46, *p* = 0.001) that were statistically significant (*p* < 0.05). No statistically significant differences were observed between the two groups with respect to education level, family income, gravidity, hypertension, and staying up late (*p* > 0.05). The detailed content is shown in Table 1.

**Table 1:** Univariate analysis of general information and clinical factors of the research subjects.

	Normal pregnancy (*n* = 9306)	Spontaneous abortion (*n* = 589)	t/χ^2^	*p*-value
**Age**	30.60 ± 5.98	33.03 ± 6.12	9.51	<0.001
BMI			9.13	0.010
≤18.4	2938	210		
18.5∼27.9	3472	184		
≥28.0	2896	195		
**Educational level**			0.01	0.917
Below the university	4672	297		
University and above	4634	292		
**Monthly household income**			0.96	0.328
<10,000 RMB	4657	307		
≥10,000 RMB	4649	282		
**Fetal number**			2.19	0.534
1	3064	177		
2	3119	197		
≥3	3123	215		
**History of embryonic arrest**			3427.87	<0.001
0	8373	68		
1	563	220		
2	345	177		
≥3	25	124		
**Number of abortions**			53.89	<0.001
0	3112	177		
1	3604	202		
2	2117	139		
≥3	473	71		
**High blood pressure**			0.03	0.867
No	8274	525		
Yes	1032	64		
**Thyroid dysfunction**			19.05	<0.001
No	9087	558		
Yes	219	31		
**Diabetes**			7.32	0.007
No	9008	558		
Yes	298	31		
**Polycystic ovary syndrome**			5.44	0.020
No	8858	548		
Yes	448	41		
**Assisted reproduction**			34.47	<0.001
No	9262	575		
Yes	44	14		
**Smoking history**			4.27	0.039
No	9181	575		
Yes	125	14		
**Drinking history**			11.62	<0.001
No	8692	571		
Yes	614	18		
**Exposure to pollution sources**			8.84	0.003
No	4743	263		
Yes	4563	326		
**Often stay up late**			1.64	0.200
No	4708	314		
Yes	4598	275		
**Recent home decoration**			10.46	0.001
No	4605	251		
Yes	4701	338		

### Univariate analysis of pregnant women's mental health

Significant differences in depression status were found between the normal pregnancy group and the spontaneous abortion group (*p* < 0.05). Technical term abbreviations have been explained upon first use. The structure is logical and causal connections between statements have been retained. British English conventions have been followed throughout, including formal register, precise word choice, and consistent citation and footnote style. In the spontaneous abortion group, a higher degree of depression was observed to be associated with a higher proportion of spontaneous abortion. The highest proportion of spontaneous abortion was observed in the group with moderate depression (31.07%), followed by severe depression (26.12%) and extremely severe depression (16.12%). The language used is clear, objective, and value-neutral, and avoids biased or ornamental expressions. Significant differences were found in anxiety levels between the normal pregnancy group and the spontaneous abortion group (*p* = 0.021). In the spontaneous abortion group, a higher degree of anxiety was associated with a greater proportion of spontaneous abortions. Moderate anxiety was the most prevalent (30.39%), followed by severe anxiety (24.82%) and extremely severe anxiety (17.44%). Significant differences in stress levels were observed between the group of women experiencing a normal pregnancy and those who had a spontaneous abortion (*p* < 0.05). The proportion of spontaneous abortions increased with the severity of stress in the latter group, with the highest rate observed in cases of severe stress (32.37%), followed by extremely severe stress (26.89%) and moderate stress (26.89%) (shown in Table 2).

**Table 2:** Single factor analysis of the mental health status of the research subjects.

	Normal pregnancy (*n* = 9306)	Spontaneous abortion (*n* = 589)	t/χ^2^	*p*-value
**Depression**			21.00	<0.001
Normal	1852	80		
Mild	1892	132		
Moderate	1883	107		
Severe	1863	126		
Extremely severe	1816	144		
**Anxiety**			11.50	0.021
Normal	1844	97		
Mild	1870	122		
Moderate	1826	146		
Severe	1871	111		
Extremely severe	1895	113		
**Stress**			20.63	<0.001
Normal	1862	77		
Mild	1835	118		
Moderate	1837	144		
Severe	1929	123		
Extremely severe	1843	127		

### Multivariate logistic regression analysis

To elucidate the effects of multiple factors on spontaneous abortion, the authors conducted a multivariate logistic regression analysis, with spontaneous abortion as the dependent variable and the aforementioned variables with significant distinctions. The study revealed a correlation between various factors and the likelihood of infertility. These factors include age (OR = 1.072, 95% CI 1.053‒1.091), a history of embryonic arrest (OR = 9.153, 95% CI 7.958‒10.528), thyroid dysfunction (OR = 8.512, 95% CI 5.273‒13.739), polycystic ovary syndrome (OR = 1.617, 95% CI 1.028‒2.543), and the use of assisted reproductive technology (OR = 12). The study found that several factors were significantly associated with spontaneous abortion, including exposure to pollution (Odds Ratio [OR] = 1.347, 95% Confidence Interval [95% CI]: 1.084‒1.674), recent home renovation (OR = 1.309, 95%CI: 1.051‒1.630), depression (OR = 1.140, 95% CI 1.055‒1.232), and stress (OR = 1.140, 95% CI 1.053‒1.233) (*p* < 0.05). However, the relationship between BMI, number of abortions, diabetes, alcohol consumption, anxiety, and spontaneous abortion was unclear (p > 0.05). The detailed content is shown in Table 3.

**Table 3:** Binary logic regression analysis of the model after adjustment.

	B	S	Wald χ^2^	p-value	OR	95%CI	
**Age**	0.069	0.009	57.819	0.000	1.072	1.053	1.091
**BMI**	-0.091	0.069	1.749	0.186	0.913	0.798	1.045
**Number of abortions**	-0.116	0.095	1.495	0.221	0.890	0.739	1.072
**History of embryonic arrest**	2.214	0.071	962.219	0.000	9.153	7.958	10.528
**Thyroid dysfunction**	2.141	0.244	76.837	0.000	8.512	5.273	13.739
**Diabetes**	-0.474	0.271	3.048	0.081	0.623	0.366	1.060
**Polycystic ovary syndrome**	1.617	0.213	57.833	0.000	5.036	3.320	7.639
**Assisted reproduction**	2.550	0.380	45.091	0.000	12.813	6.086	26.975
**Smoking history**	1.007	0.372	7.338	0.007	2.739	1.321	5.676
**Drinking history**	0.287	0.288	0.997	0.318	1.333	0.758	2.344
**Exposure to pollution sources**	0.298	0.111	7.211	0.007	1.347	1.084	1.674
**Recent home decoration**	0.269	0.112	5.784	0.016	1.309	1.051	1.630
**Depression**	0.131	0.040	10.900	0.001	1.140	1.055	1.232
**Anxiety**	-0.028	0.039	0.497	0.481	0.973	0.900	1.051
**Stress**	0.131	0.040	10.497	0.001	1.140	1.053	1.233

### Model development

Nine predictors were included in the final model based on clinical relevance and statistical significance on multivariate analysis (*p* < 0.05): maternal age, history of embryonic arrest, thyroid dysfunction, polycystic ovary syndrome, assisted reproduction, exposure to pollution, recent home renovation, depression score, and stress score.

The final prediction model equation is: Risk of spontaneous abortion = 1/(1 + e^-(Y)) Where Y = -3.283 + 0.069 × Age + 2.352 × Embryonic arrest + 1.954 × Thyroid dysfunction + 0.479 × PCOS + 2.996 × ART + 0.245 × Pollution + 0.237 × Renovation + 0.131 × Depression score + 0.125 × Stress score.

Age is in years, PCOS is polycystic ovary syndrome (1 = present, 0 = absent), ART is assisted reproduction (1 = used, 0 = not used), Pollution is exposure to pollution (1 = exposed, 0 = not exposed), Renovation is recent home renovation (1 = renovation, 0 = no renovation). The other variables are continuous measures.

The exponentiated coefficients represent the odds ratio for each predictor variable. For example, the odds of spontaneous abortion increase by 1.069 times for each 1-year increase in maternal age. This full model equation allows the calculation of predicted risks of spontaneous abortion for individual patients based on their predictor values. It can be incorporated into a nomogram, web calculator, or mobile app to obtain predicted risks.

### Model performance

**Fig. 2:**
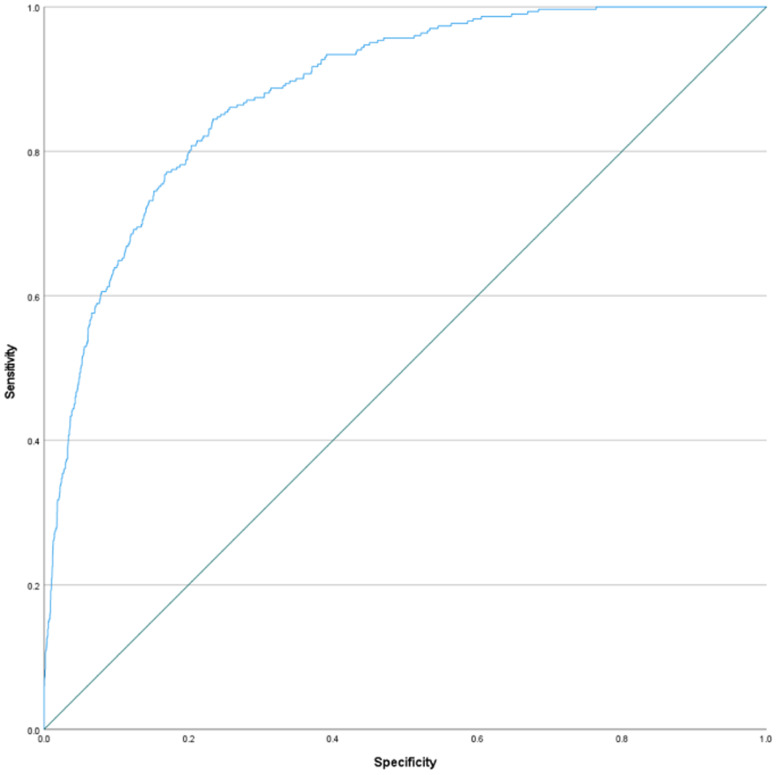
Receiver Operating Characteristic (ROC) curve for discrimination of early spontaneous abortion.

The discrimination of the model was excellent with a C-statistic of 0.88 (95% CI 0.87–0.90). The C-statistic indicates the ability of the model to differentiate between patients who did and did not experience a spontaneous abortion (Fig. 2). After internal validation with bootstrapping, the optimism-adjusted C-statistic was 0.87, indicating minimal overfitting.

Calibration refers to how closely the predicted risks agree with observed risks. The calibration plot showed good agreement between predicted and observed spontaneous abortion risks across tenths of predicted risk. The Hosmer-Lemeshow test also demonstrated good calibration (*p* = 0.27).

A predicted probability threshold of >0.08 was selected based on the Youden index to optimize the balance of sensitivity and specificity. The model classified 6.5% of patients as high risk using a predicted probability threshold of >0.08. Among these women, the observed spontaneous abortion rate was 12.4%, compared to 4.7% in the low-risk group. The sensitivity and specificity were 72% and 84%, respectively. The negative predictive value was 97%, suggesting the model was very effective at identifying women at low risk of spontaneous abortion.

## Discussion

This study developed and validated a clinical prediction model for estimating first-trimester spontaneous abortion risk in Chinese women, demonstrating good discrimination and calibration. The model enables individualized risk assessment based on a multitude of demographic, clinical, lifestyle and mental health predictors. With further validation, it holds promise to guide counseling and interventions for high-risk women.

Several robust predictors emerged, including advanced maternal age, obstetric history, chronic conditions like thyroid disorders and polycystic ovary syndrome, assisted reproduction, toxic environmental exposures, and poor mental health.^8^, ^9^ The wide range of factors underscores the complex multifactorial etiology of spontaneous abortion.^10^

Advanced age likely contributes through age-related reductions in oocyte quality, uterine receptivity, and embryo aneuploidy.^11^ Recurrent pregnancy loss may reflect cumulative damage to endometrial function and the maternal-fetal interface.^12^ Medical comorbidities such as thyroid disease can perturb the hormonal milieu and metabolic environment needed to sustain early pregnancy.^13^^,^^14^ Assisted reproduction increases risks due to underlying subfertility, and effects of controlled ovarian stimulation and laboratory procedures.^15^ Environmental toxins can disrupt placental development and trigger embryonic oxidative stress and DNA damage.^16^, ^17^ Psychological distress may impact uterine blood flow, inflammation, cortisol, and immune balance.^18^, ^19^, ^20^, ^21^

This study has several strengths. Firstly, the large prospective cohort allowed for the analysis of numerous candidate variables. Secondly, rigorous adherence to TRIPOD guidelines enhanced model development and internal validation. Finally, discrimination and calibration metrics indicate good predictive performance.

Limitations of this study include its single-center design and reliance on self-reported data, which may introduce residual confounding given the observational design. Furthermore, external validation and impact analysis are required prior to clinical application of the model. Future refinements incorporating emerging biomarkers and modifiable risk factors may further enhance its utility.

## Conclusion

This study highlights that spontaneous abortion susceptibility is influenced by a complex interplay of maternal age, obstetric history, chronic medical conditions, mental health, and environmental factors. The prediction model enables individualized risk quantification to guide the management of high-risk women. With ongoing validation and refinement, it has significant potential to optimize outcomes and reduce the burden of this common pregnancy complication. A multidimensional approach addressing medical, psychological, and environmental health is recommended for optimal management of spontaneous abortion susceptibility.

## Data availability statements

The data that support the findings of this study are available from the corresponding author upon reasonable request. The data are not publicly available due to privacy or ethical restrictions.

## Authors’ contributions

Xuetao Hou and Min Lv conceived and designed the study. Jimei Yang and Zhijing Chen collected data. Xiang Wang and Zhen Song analyzed the data. Junqing Li and Jimei Yang drafted the manuscript. All authors have reviewed and approved the final version of the manuscript prior to submission.

## Funding

This study was supported by the 2023 Jinan Municipal Health Commission Big Data Technology Plan Project (2023-YBD-1-05).

## Declaration of Competing Interest

The authors declare no conflicts of interest.
